# Supplementary material for: PSEN1 His214Asn Mutation in a Korean Patient with Familial EOAD and the Importance of Histidine–Tryptophan Interactions in TM-4 Stability
Source: Int J Mol Sci. 2023 Dec 21;25(1):116. doi: 10.3390/ijms25010116 (PMC10778985; doi:10.3390/ijms25010116)

Supplementary table S1. Mutations, found in the patient

Gene_Name	HGVS.c	HGVS.p	Zygosity	rs ID	p3_1000G	p3_1000G_EAS_AF	gnomAD_exomes_AF	gnomAD_exomes_EAS_AF	SIFT_score	SIFT_pred
ABCA13	c.1517C>T	p.Pro506Leu	HET	rs1880738	0.40655	0.6181	0.449307	0.575825	0.436	T
	c.4618T>C	p.Phe1540Leu	HET	rs17712299	0.096446	0.1498	0.07085	0.179048	0	D
	c.6533C>A	p.Ala2178Glu	HOM	rs1880736	0.812899	0.9881	0.723538	0.989164	1	T
	c.6917A>C	p.Lys2306Thr	HET	rs78334925	0.097244	0.1508	0.075345	0.186615	0.11	T
	c.8020C>T	p.Arg2674Trp	HOM	rs2222648	0.852236	0.9921	0.803456	0.991853	1	T
	c.11123A>T	p.Gln3708Leu	HET	rs143050255	0.002995	0.0149	0.001078	0.014851	0.028	D
	c.12829A>G	p.Asn4277Asp	HET	rs4917152	0.230831	0.2163	0.172627	0.239183	1	T
	c.12905A>G	p.Gln4302Arg	HET	rs4917153	0.230631	0.2163	0.172591	0.239214	0.64	T
	c.13949A>G	p.Glu4650Gly	HET	rs557105317	0.0002	0.001	3.66E-05	0.000524	0.032	D
ABCA7	c.563A>G	p.Glu188Gly	HET	rs3764645	0.399561	0.4355	0.497496	0.428977	0.647	T
	c.1388G>A	p.Arg463His	HET	rs3752233	0.060703	0.1915	0.046649	0.176792	0.254	T
	c.2153A>C	p.Asn718Thr	HET	rs3752239	0.059105	0.1855	0.047099	0.16968	0.239	T
	c.4046G>A	p.Arg1349Gln	HET	rs3745842	0.390575	0.3482	0.427897	0.335604	0.546	T
	c.4580G>C	p.Gly1527Ala	HOM	rs3752246	0.825479	0.6478	0.840229	0.646508	0.877	T
	c.5057A>G	p.Gln1686Arg	HET	rs4147918	0.05651	0.1736	0.04749	0.1651	0.234	T.

	c.6133G>T	p.Ala2045Ser	HET	rs4147934	0.605032	0.4325	0.71193	0.446435	0.962	T.
ACOX1	c.936C>G	p.Ile312Met	HOM	rs1135640	0.550719	0.7877	0.653791	0.800441	0.256	T
ADAM18	c.1883C>T	p.Thr628Ile	HET	rs183758075	0.000599	0.003	0.000233	0.002922	0.286	T
ALS2	c.1102G>A	p.Val368Met	HOM	rs3219156	0.896565	1	0.912953	0.999826	0.191	T
ARAP2	c.4568G>A	p.Arg1523Gln	HOM	rs4833069	0.995208	1	0.992128	1	1	T
ATP13A2	c.3214G>A	p.Ala1072Thr	HOM	rs3170740	0.33127	0.246	0.47141	0.295664	0.988	T
ATXN1	c.672_677delGCAGCA	p.Gln224_Gln225del	HET	rs754954093	.	.	.	.	.	.
	c.647_648insTCA	p.Gln215_Gln216insHis	HET	rs766029394	.	.	.	.	.	.
	c.642G>T	p.Gln214His	HET	rs200111316	.	.	.	.	0.581	T
	c.639G>T	p.Gln213His	HET	rs3817753	.	.	.	.	0.072	T
	c.633T>G	p.His211Gln	HET	rs59310777	0.266374	0.3879	.	.	0.297	T
	c.627_629delTCA	p.His209del	HET	rs751377396	.	.	.	.	.	.
	c.627T>G	p.His209Gln	HET	rs11969612	.	.	.	.	0.065	T
ATXN2	c.2285A>T	p.Asn762Ile	HET	rs191400641	0.001597	0.0079	0.00079	0.011027	0.077	T
	c.743G>A	p.Ser248Asn	HOM	rs7969300	0.179513	0.5228	0.076308	0.564288	0.136	T
	c.563_565delAGC	p.Gln188del	HOM	rs10560189;rs67658094	0.951078	1	.	.	.	.
BST1	c.434G>A	p.Arg145Gln	HET	rs2302464	0.079074	0.2232	0.047079	0.208164	0.111	T

CASS4	c.1978C>T	p.Pro660Ser	HOM	rs35031530	0.146765	0.2381	0.048838	0.254697	0.286	T
CD33	c.41C>T	p.Ala14Val	HET	rs12459419	0.210663	0.1855	0.308116	0.184375	0.029	D
COL4A1	c.4002A>C	p.Gln1334His	HET	rs3742207	0.288339	0.2629	0.304165	0.251565	0.122	T
	c.19G>C	p.Val7Leu	HET	rs9515185	0.424121	0.6161	0.435687	0.633385	0.575	T
CR1	c.5573C>T	p.Thr1858Met	HET	rs3737002	0.248802	0.3264	0.282939	0.331399	0.019	D
	c.6178A>T	p.Thr2060Ser	HOM	rs4844609	0.995008	1	0.985426	1	0.804	T
	c.7255A>G	p.Thr2419Ala	HET	rs2296160	0.828075	0.6885	0.819882	0.655775	0.987	T
CTSA	c.108_110delGCT	p.Leu37del	HET	rs1457838268	0.649361	0.5784	.	.	.	.
DDX1	c.1620C>G	p.Phe540Leu	HET	rs201143228	.	.	4.10E-05	0	0.267	T
DNMBP	c.4239T>G	p.Cys1413Trp	HET	rs11190305	0.277157	0.244	0.344252	0.238694	0.181	T
	c.1143C>G	p.Ser381Arg	HET	rs7077718	0.486821	0.2569	.	.	.	.
DSG2	c.1984G>A	p.Ala662Thr	HET	rs1186896680	.	.	4.07E-06	0	0	D
	c.2318G>A	p.Arg773Lys	HET	rs2278792	0.240016	0.4643	0.265563	0.466526	0.383	T
EPHA1	c.2698A>G	p.Met900Val	HOM	rs6967117	0.960264	1	0.940039	0.998493	1	T
	c.479T>C	p.Val160Ala	HOM	rs4725617	0.94349	0.9921	0.93221	0.991984	0.246	T
	c.71G>A	p.Arg24His	HET	rs79587607	0.086062	0.3006	.	.	0.149	T
FBXO7	c.35T>G	p.Leu12Arg	HOM	rs8137714	0.154952	0.1567	0.190958	0.143187	0.258	T
	c.3G>A	p.Met1?	HOM	rs11107	0.48742	0.6915	.	.	.	.

FIG4	c.1090A>T	p.Met364Leu	HET	rs2295837	0.10004	0.1984	0.07893	0.207343	1	T
FNDC1	c.1312A>G	p.Thr438Ala	HET	rs509648	0.508586	0.7232	0.324572	0.751479	1	T
	c.1387G>C	p.Glu463Gln	HET	rs420137	0.784545	0.621	0.866743	0.637705	1	T
	c.3007C>G	p.Gln1003Glu	HET	rs370434	0.785743	0.626	0.867677	0.641117	1	T
	c.3540C>G	p.Asp1180Glu	HET	rs420054	0.782348	0.626	0.850326	0.622257	1	T
	c.3782T>C	p.Leu1261Pro	HET	rs3003174	0.786142	0.626	0.86882	0.641139	0.345	T
	c.3839A>G	p.Gln1280Arg	HET	rs2501176	0.786142	0.626	0.869494	0.643025	1	T
	c.4436_4453delCCCGCCGCACGACCACCA	p.Thr1479_Thr1484del	HET	rs141435210;rs3842694	0.496406	0.4058	.	.	.	.
	c.4511C>A	p.Thr1504Lys	HET	rs386360	0.784944	0.625	0.872142	0.644028	0.795	T
	c.4720A>G	p.Thr1574Ala	HET	rs7763726	0.145567	0.3552	0.064727	0.318933	0.042	D
	c.1139_1141dupGCG	p.Gly380dup	HOM	rs545470261;rs76840944	0.152157	0.2867	.	.	.	.
FOXF2	c.121_123dupGCC	p.Ala41dup	HET	rs752348150	.	.	.	.	.	.
	c.917_919dupGCG	p.Gly306dup	HOM	rs147426137;rs397731476;rs58230522	0.392772	0.497	.	.	.	.
GAB2	c.404G>T	p.Gly135Val	HET	rs76853537	0.011981	0.001	0.012685	0.002319	0.534	T
GIGYF2	c.1441C>A	p.Pro481Thr	HOM	rs2289912	0.083067	0.2302	0.054389	0.224298	0.133	T
	c.3692_3693insGC	p.Gln1232fs	HOM	rs371622656	.	.	.	.	.	.
	c.3693_3694insG	p.Gln1232fs	HOM	rs775324034	.	.	.	.	.	.

HIP1R	c.1873C>T	p.Arg625Trp	HET	rs117866676	0.014177	0.0685	0.00527	0.066624	0.036	D
HTRA1	c.33_34insT	p.Leu12fs	HET	rs541533723	.	.	.	.	.	.
	c.34_35insAT	p.Leu12fs	HET	.	.	.	.	.	.	.
INPP5D	c.3505C>T	p.His1169Tyr	HOM	rs9247	0.240016	0.3482	0.218841	0.364107	0.119	T
LPA	c.5673A>G	p.Ile1891Met	HET	rs3798220	0.051318	0.0883	0.055489	0.08888	0.116	T
	c.5036T>C	p.Met1679Thr	HET	rs1801693	0.648163	0.4494	0.642298	0.451334	1	T
	c.4114C>G	p.Leu1372Val	HET	rs7765781	0.414936	0.4107	0.355312	0.396864	0.967	T
	c.4072C>G	p.Leu1358Val	HET	rs7765803	0.409145	0.4107	0.353439	0.397132	1	T
LRRK2	c.149G>A	p.Arg50His	HOM	rs2256408	0.969249	1	0.993066	1	1	T
	c.3857A>G	p.Asn1286Ser	HET	rs777961379	.	.	2.44E-05	0	0.038	D
	c.4939T>A	p.Ser1647Thr	HOM	rs11564148	0.285942	0.3373	0.298352	0.33871	0.953	T
	c.7173C>G	p.His2391Gln	HET	rs199680004	.	.	6.93E-05	0.000986	0.43	T
	c.7190T>C	p.Met2397Thr	HOM	rs3761863	0.551717	0.4603	0.618476	0.46794	0.466	T
MAPT	c.1321T>C	p.Tyr441His	HOM	rs2258689	0.312899	0.628	0.281649	0.632031	0.978	T
NME8	c.128G>A	p.Arg43Lys	HET	rs2722372	0.245208	0.1815	0.228742	0.184027	1	T
	c.440C>T	p.Pro147Leu	HET	rs143559107	0.000998	0.005	0.000285	0.003888	0.309	T
	c.622T>C	p.Cys208Arg	HOM	rs10250905	0.743411	0.5466	0.731553	0.560475	0.046	D
	c.1478T>C	p.Ile493Thr	HET	rs56128139	0.157748	0.1359	0.27337	0.128381	0.032	D

NOTCH3	c.709G>A	p.Val237Met	HET	rs2285981	0.000799	0.004	0.000195	0.002436	0.002.	D
NOTCH4	c.1315G>C	p.Ala439Pro	HET	rs200731043	0.0002	0.001	0.000756	0.010785	0.012	D
	c.349A>C	p.Lys117Gln	HOM	rs915894	0.398762	0.5109	0.351497	0.46229	0.399	T
	c.45_47delGCT	p.Leu16del	HOM	rs35795312	0.47524	0.4921	.	.	.	.
PDLIM5	c.41C>T	p.Ser14Phe	HOM	rs2452600	0.224641	0.378	0.265912	0.374971	0.024.	D
	c.1120G>A	p.Ala374Thr	HOM	rs966845	0.993411	1	0.993729	1	0.709	T
	c.1562G>A	p.Ser521Asn	HOM	rs13107595	0.979633	1	0.989681	1	0.91	T
PSEN1	c.640C>A	p.His214Asn	HET	.	.	.	.	.	0.001	D
PTK2B	c.2513A>C	p.Lys838Thr	HET	rs751019	0.364617	0.3601	0.440573	0.343982	0.439	T
RIN3	c.331G>A	p.Glu111Lys	HET	rs2274542	0.022364	0.0595	0.012089	0.077967	0.31	T
	c.2913_2915delCGG	p.Gly972del	HET	rs570458246;rs68153141;rs71698059	.	.	.	.	.	.
SACS	c.10106T>C	p.Val3369Ala	HOM	rs17078605	0.258986	0.3145	0.286729	0.350505	0.014	D
SETX	c.7834A>G	p.Ser2612Gly	HET	rs3739927	0.163538	0.3591	0.084295	0.387787	0.652	T
	c.7759A>G	p.Ile2587Val	HOM	rs1056899	0.538738	0.6835	0.38054	0.728828	1	T
	c.5563A>G	p.Thr1855Ala	HOM	rs2296871	0.443091	0.6379	0.263905	0.696578	0.83	T
	c.3455T>G	p.Phe1152Cys	HET	rs3739922	0.089657	0.2371	0.064566	0.269655	0.003	D
	c.1979C>G	p.Ala660Gly	HOM	rs882709	0.213658	0.4117	0.115986	0.44578	0.008	D
SFRP4	c.958C>A	p.Pro320Thr	HET	rs1802073	0.542732	0.5635	0.435974	0.550848	0.171	T

SIGMAR1	c.5A>C	p.Gln2Pro	HET	rs1800866	0.217252	0.3204	0.202067	0.314972	0.343	T
SLC24A4	c.1654A>C	p.Lys552Gln	HET	rs45587635	0.067292	0.1796	0.059926	0.228744	0.184	T
SLC6A5	c.304G>A	p.Gly102Ser	HOM	rs1443547	0.405751	0.3651	0.368835	0.393579	0.723	T
	c.371T>C	p.Phe124Ser	HOM	rs1443548	0.793131	0.75	0.771566	0.760212	0.44	T
	c.485C>G	p.Ala162Gly	HOM	rs1443549	0.99361	1	0.998607	1	1	T
SMC5	c.916G>A	p.Val306Ile	HOM	rs1180116	0.855032	0.8532	0.88404	0.855722	0.655	T
SORL1	c.3220C>G	p.Gln1074Glu	HOM	rs1699107	0.984824	1	0.996113	1	0.168	T
	c.5899G>A	p.Val1967Ile	HOM	rs1792120	0.979433	1	0.996169	1	1	T
STK39	c.661G>A	p.Gly221Ser	HET	rs200257510	0.0002	0.001	9.83E-05	0.001113	0.07	T
SYT11	c.144G>C	p.Gln48His	HOM	rs822522	0.954673	1	0.990874	0.999942	0.866	T
TET1	c.485A>G	p.Asp162Gly	HOM	rs10823229	0.258586	0.3919	0.330584	0.383148	0.019	D
	c.3369A>G	p.Ile1123Met	HOM	rs3998860	0.693291	0.8502	0.777419	0.839626	0.157	T
TM2D3	c.17T>G	p.Leu6Arg	HOM	rs2939587	0.993411	1	0.97935	1	0.311	T
TOMM40	c.934C>G	p.Leu312Val	HET	rs1275991853	.	.	.	.	0.307	T
VEPH1	c.1564T>C	p.Ser522Pro	HET	rs11918974	0.250599	0.3512	0.276265	0.392621	1	T
ZCWPW1	c.628G>C	p.Ala210Pro	HET	rs768069661	.	.	8.13E-06	0.000116	0.003	D

Supplementary figures.

Supplementary figure S1. Structure predictions of PSEN1 mutations in His214 residue. (a) Normal PSEN1 (b) PSEN1 His214Asp (c) His214Arg (d) His214Tyr

Structure prediction of PSEN1 His214 other mutations. His214Asp seems to have similar effect like His214Asn, since it could also result in the loss of interaction between His214 and Trp215. His214Asp seems to have similar effect like His214Asn, since it could also result in the loss of interaction between His214 and Trp215. Due to its aromatic ring, Tyr214 may also interact with Trp215. The mutation may not significantly change the intramolecular interactions. However, histidine is a positively charged residue while tyrosine is a hydrophobic one, which may change the helix dynamics or result in putative abnormal intermolecular interactions.

Supplementary figure S1. Structure predictions of PSEN1 mutations in His214 residue. (a) Normal PSEN1 (b) PSEN1 His214Asp (c) His214Arg (d) His214Tyr

a. **b.** **c.** **d.**

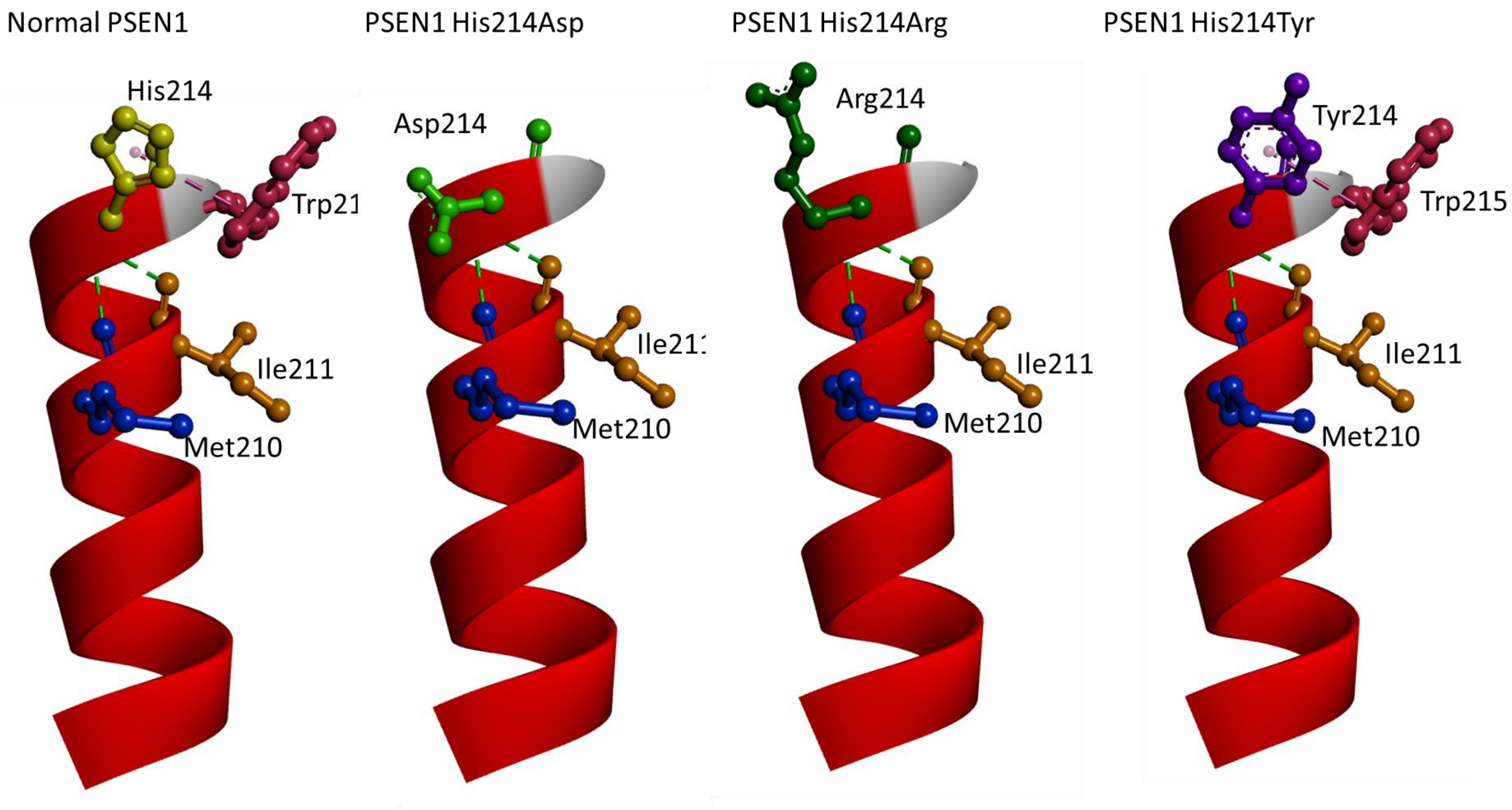

Supplement: Supplementary file 1 [file ijms-25-00116-s001.zip › ijms-2769595-supplementary.pdf]
